# Particle-Size-Determined Crystallization and Dissolution Behavior of Amorphous Griseofulvin

**DOI:** 10.1007/s11095-025-03984-3

**Published:** 2025-12-10

**Authors:** Daniela Košťálová, Roman Svoboda, Kateřina Kozlová, Marie Nevyhoštěná, Alena Komersová

**Affiliations:** Department of Physical Chemistry, Faculty of Chemical Technology, University of Pardubice, Studentská 573, 532 10 Pardubice, Czech Republic

**Keywords:** Amorphous griseofulvin, Crystalline griseofulvin, Crystallization, Dissolution, DSC, Griseofulvin powder fraction, Kinetic analysis, Particle size

## Abstract

**Purpose:**

Amorphous active pharmaceutical ingredients (APIs) are generally considered to have significantly higher bioavailability, compared to their crystalline counterpart, due to the enhanced solubility of the disordered phase. However, an akin functionality can be also adopted by the particle size of the powdered API. In this case study, a detailed investigation of the particle-size-influenced properties of amorphous griseofulvin powders will be introduced.

**Methods:**

The crystallization of amorphous griseofulvin powders in the range 20 – 1000 μm (+ 2 – 10 μm only for crystalline form) was studied calorimetrically, spectroscopically, and microscopically. Dissolution profiles of pharmaceutical tablets with incorporated either amorphous or crystalline griseofulvin were obtained under conditions simulating the path through the gastrointestinal tract.

**Results:**

Standard crystal growth regime was accompanied by the rapid diffusionless growth mode, which was detected at low heating rates for the finest griseofulvin powders. The dissolution profiles of the pharmaceutical tablets with incorporated individual griseofulvin powder fractions were described in terms of the Korsmeyer-Peppas model (indicating the release by super case II transport).

**Conclusion:**

Particle size was found to play dominant role in the dissolution kinetics, whereas the difference in the dissolution rates of the crystalline and amorphous particles was rather negligible. This is a beneficial finding, considering the very low stability of finely powdered amorphous griseofulvin, but at the same time, it negates the primary purpose of amorphization. Main benefit is thus that of the coarse amorphous griseofulvin powder, which can be utilized to fine-tune the dissolution profile due to its delayed dissolution.

## Introduction

The present article deals with an active pharmaceutical ingredient (API), griseofulvin (GSF), which is used to treat fungal skin, nail, and hair diseases. Also, griseofulvin has recently been confirmed to have antiproliferative activity in various types of cancer cells (tumor growth inhibition) in athymic mice [1–4].

The griseofulvin is an API belonging to the second (II) class of the Biopharmaceutical Classification System (BCS). It means that GSF has low solubility and high permeability [5]. The first crystal structure, i.e., the most stable form (form I) of the griseofulvin, was described already in 1977. However, the subsequent attempts to obtain other polymorphs using the crystallization from a solution still yielded the already known crystalline form (form I). For several decades, this API was considered monomorphic, i.e., it existed only in one crystal structure. In a recent study [6], the two new polymorphic forms using melt crystallization (form II and form III) were discovered and characterized by X-ray diffraction (XRD) and differential scanning calorimetry (DSC). Although the found forms are metastable compared to form I, they are long-lived at room temperature. The solid-state of the API can play an important role in the bioavailability, given that metastable phases can show higher solubility than the stable form [5–7].

To predict the crystallization behavior of the amorphous griseofulvin under various experimental, storage, or production conditions and to assess its long-term physical stability, it is necessary to know the base thermal properties as well as the advanced kinetics of the crystallization of the griseofulvin. For this purpose, various methods can be used. The crystallization kinetics of the griseofulvin can be investigated using differential scanning calorimetry (DSC). The kinetics of the griseofulvin decomposition (crucial, e.g., for the determination of the temperature limits for the hot-melt extrusion and 3D-printing processing) can be investigated using thermogravimetry (TG). The crystallization and the crystallization mechanism of the amorphous griseofulvin can also be investigated using optical microscopy or scanning electron microscopy (SEM). For the pharmaceutical industry, the investigation of the absorption of the API in the human body (*in vivo*) is crucial. In this regard, the dissolution test can be chosen as an eligible tool due to its ability to predict the absorption of the drug and determine the mechanism by which the API is released from the dosage form [8–13].

In the present article, a novel approach to the design of the amorphous API-based drugs will be discussed, focusing on the influence of different particle sizes of the amorphous grains. In particular, a multi-topic investigation interconnecting the solid-state characterization with the consequent dissolution profiles will be performed, utilizing the above-mentioned methods to explore the particle-size-resolved crystallization behavior of amorphous griseofulvin and to quantify the effects of the powder micronization on the dissolution profile. Whereas the latter is explored in the literature [14–16], only few studies actually compare the crystalline and amorphous data in a wide range of particle sizes in conjuction of being incorporated into a pharmaceutically relevant controlled-release polymeric matrix, where the API tendency toward crystallization (defined by particle size and determined independently) may play a crucial role in (de)stabilization of the amorphous phase during the dissolution – a feature known, e.g., for amorphous indomethacine [17].

## Experimental

### The Preparation of the Amorphous Griseofulvin

#### The Melt-Quench Preparation of the Amorphous Griseofulvin

The amorphous griseofulvin was obtained by the melt-quench technique. For this purpose, the powdered crystalline griseofulvin (Sigma Aldrich, from Penicillium griseofulvum, 97.0–102.0%) was used. The crystalline griseofulvin was transferred to a fused silica ampoule so that the ampoule was filled with powdered crystalline griseofulvin to a height of approx. 2 cm. The ampoule with the griseofulvin was placed in a container with oil, which was heated above the melting point of griseofulvin, i.e., 220–230 °C. After the crystalline phase was completely melted, the ampoule containing the griseofulvin melt was removed from the container and rapidly cooled in cold water.

#### The Sieving Procedure of the Prepared Amorphous Griseofulvin

The ampoule containing the amorphous ingot was broken, and the glassy griseofulvin pieces were broken (by gentle tapping) into small powder grains in an agate mortar. The true grinding was reduced to a minimum so that no mechanically induced crystallization could occur due to the large evolution of the friction heat. The amorphous powder was split into several fractions using sieves with a defined mesh size. The following powder fractions of the amorphous griseofulvin were prepared: 20–50 μm, 50–125 μm, 125–180 μm, 180–250 μm, 250–300 μm, 300–500 μm, and 500–1000 μm.

### Differential Scanning Calorimetry (DSC)

The amorphous GSF powders were examined using the heat-flow differential scanning calorimeter DSC Q2000 (TA Instruments) equipped with an RCS90 cooling device, T-zero technology, and an autosampler (ensuring a rapid continuous course of the DSC measurements, completing the whole part of the study within few days). The GSF powders were analytically weighed (2—3 mg; precision of ± 0.01 mg) into the aluminum crucibles and spread on the bottom of the crucible to ensure the best possible thermal contact. The aluminum crucibles containing samples were hermetically sealed and measured non-isothermally in the 10–250 °C temperature range. The applied heating rates q^+^ were 0.5, 1, 2, 5, 10 and 20 °C·min^−1^.

### Thermogravimetry (TG)

The prepared samples were also examined by the TG, specifically using the NETZSCH instrument (STA 449 F5 Jupiter). The sample masses ranged from 2 to 3 mg (again, accurately weighted to ± 0.01 mg). The measurements were performed non-isothermally in the temperature range of 30–400 °C at q^+^  = 10 °C·min^−1^; two types of atmosphere (N_2_ and dry synthetic air) were used during two separate measurements.

### Raman Spectroscopy

The Raman spectra for the amorphous, partially and fully crystalline GSF powders were collected using the DXR2 Raman spectroscope (Nicolet, ThermoFisher) equipped with a 785 nm laser (power of 25 mW; spot size of 3 μm). The overall collection times ranged between 10–100 s, depending on the nature of the sample.

### Microscopic Analysis

#### Optical Microscopy

Microscopic analyses were done for the samples prepared by a quench (via metal heat sink) of a griseofulvin droplet on a microscopic slide. The surface of the frozen-in droplets was gently touched by tweezers to induce mechanical damage (micro-scratches or cracks). Otherwise, no crystalline phase would be formed on a smooth surface. For the optical microscopy, the sample was shortly nucleated at 85 °C and then left for 30 days at laboratory temperature. The optical microscopy observations were performed with the iScope PLMi (Euromex) equipped with objectives with a magnification range of 4x—80x.

#### Scanning Electron Microscopy (SEM)

The sample for the SEM measurements was partially crystallized (20 min; 90 °C) and immediately measured. The TM4000 Plus II (Hitachi) scanning electron microscope equipped with the back-scattered electron detector was used; the morphology of the different layers of the studied sample was examined at an accelerating voltage of 10 kV and at 2000 × and 3000 × magnifications.

### Dissolution Analysis

#### Tablet Manufacturing Procedure

In the case of the dissolution test, the tablets with a hydrophilic matrix were prepared from the original crystalline powdered griseofulvin and from the individual fractionalized amorphous powders. Six tablets from the powdered crystalline griseofulvin and six tablets from each amorphous fraction were prepared by the direct compression method. Also, one blank tablet was prepared for each dissolution, where the given amount of the griseofulvin was replaced by the dry binder Prosolv ® SMCC 90. The composition of the tablets is shown in Table 1; the weight of each tablet was 500 mg ± 5 mg. Needed substances were mixed in a mixer (RETSCH MM200, Retsch, Haan, Germany). Then the mixed material was directly compressed on a 13 mm flat punch set using a manual single-punch tablet press (H-62- TRYSTOM spol. s.r.o., Olomouc, Czech Republic). The obtained tablets had a cylindrical shape without facets with a diameter of 13 mm and a weight of 0.5 ± 0.0010 g. All substances were accurately analytically homogenized. The conditions of homogenization were following, the homogenization rates of 10, 13, and 15 vibrations per second were used and each used homogenization rate operated with a duration of 60 s. The homogenized powder was transferred to a press unit with a diameter of 13 mm and maintained for 5 min. at a compression force of 8 kN, which relates to a compression pressure of 45.294 MPa for the obtained cylindrical tablets with a 13 mm diameter.

#### Dissolution Method

The SOTAX AT 7 Smart dissolution apparatus (SOTAX Pharmaceutical Testing s.r.o.) was used to perform the paddle dissolution method. The dissolution media with a pH of 1.2 and 6.8 were used to simulate the pH values ​​in the parts of the human gastrointestinal tract (GIT), specifically in the stomach (pH 1.2) and in the intestine region (pH 6.8); the media were prepared in accordance with European Pharmacopoeia [18].

##### Preparation of a Dissolution Medium With pH 1.2 (Gastric Region of Human GIT)

A two-liter volumetric flask was used to prepare a dissolution medium with a pH value of 1.2, into which 500 mL of a 0.2 M sodium chloride (NaCl) solution and 850 mL of a 0.2 M hydrochloric acid (HCl) solution were quantitatively transferred. This solution was then supplemented with redistilled water to a total volume of 2 l.

##### Preparation of a Dissolution Medium With pH 6.8 (Intestine Region of Human GIT)

A two-liter volumetric flask was also used to prepare a dissolution medium with a pH of 6.8, into which 500 mL of a 0.2 M potassium dihydrogen phosphate solution (KH_2_PO_4_) and 224 mL of a 0.2 M sodium hydroxide solution (NaOH) were quantitatively transferred. This solution was supplemented with redistilled water to a total volume of 2 l [19].

### Dissolution Tests

The series of dissolution tests were performed always with one blank tablet and six tablets with a given size fraction of the griseofulvin. The 900 mL of the dissolution medium with a pH value of 1.2 was put into the individual vessels that were placed in the apparatus; after reaching the temperature of the medium to 37 °C ± 0.5 °C, the sample tablets were thrown in, and the dissolution program was set. After 2 h at pH = 1.2, the medium was quickly sucked out of the container and immediately replaced by a pre-tempered medium with pH = 6.8, in which the dissolution continued for another 22 h. The stirring frequency was 100 rev·min^−1^. The samples of the medium with (potentially) dissolved API were automatically collected at progressively increasing times and analyzed by UV/VIS spectrometry (wavelength 295 nm). The absorbance (measured at the absorption maximum corrected via a three-point approximation of the background) was always measured against the blank tablet; the amounts of the released API were calculated based on the calibration curve method.

## Results and Discussion

The present section will be divided into three parts. First, the base solid-state characterization data produced for the prepared amorphous griseofulvin powders will be introduced. Namely, the findings obtained by thermoanalytical, spectroscopic, and microscopic techniques will be discussed. In the second section, the particle-size-resolved crystallization kinetics will be described in detail, stressing the impact of the particle size on the crystallization tendency. The third section will then focus on the dissolution behavior of individual amorphous powders; a comparison with the very finely milled crystalline griseofulvin powder will be discussed again in relation to the particle size and higher crystallization tendency of fine amorphous GSF powders (potentially) influencing the dissolution rate.

### Base Solid-State Characterization of Amorphous Griseofulvin Powders

#### DSC and Raman Spectroscopy Characterization

**Fig. 1:**
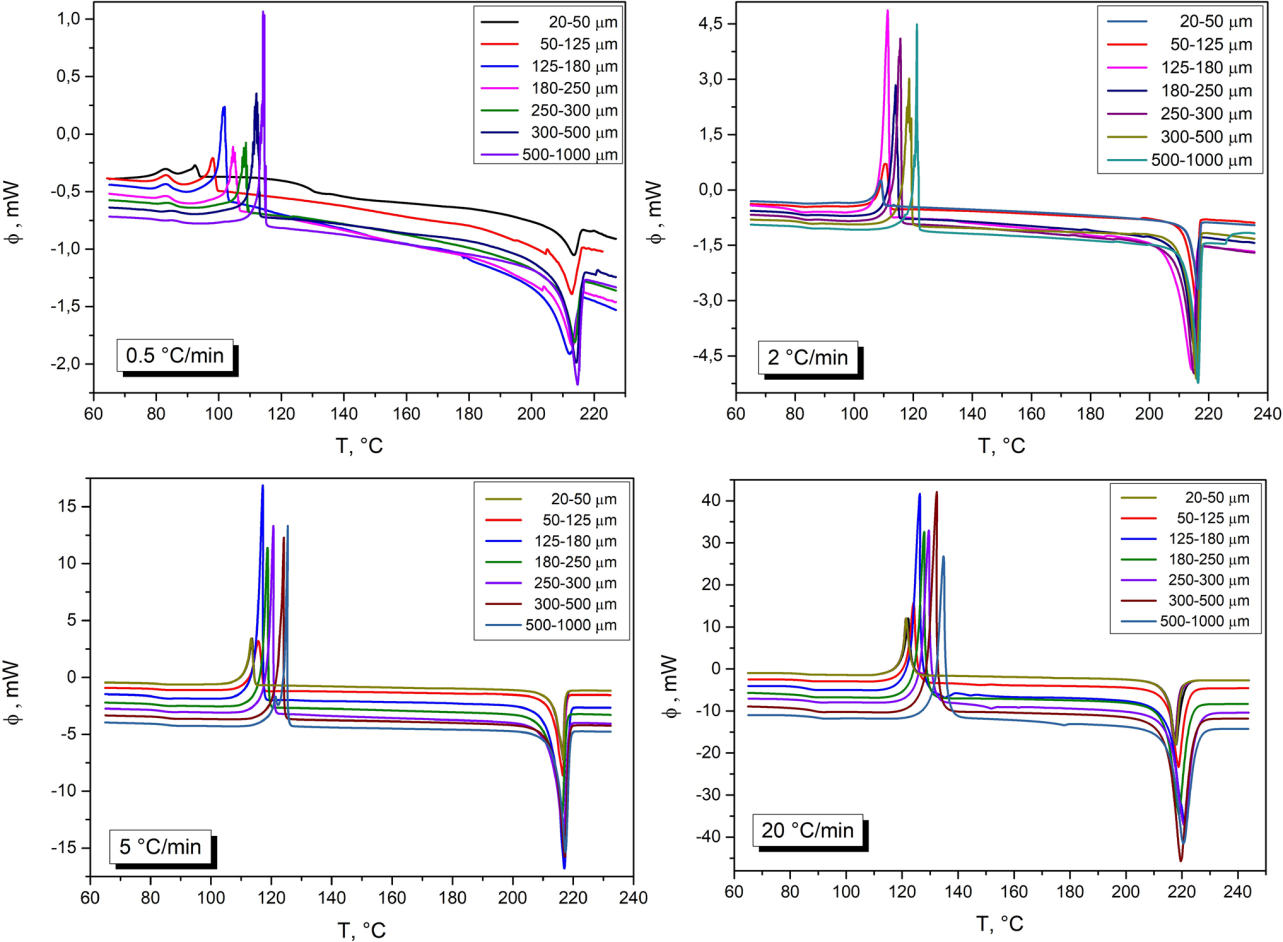
The DSC curves obtained for all studied particle size fractions and chosen applied heating rates. The DSC curves for the lowest applied heating rate (q^+^  = 0.5 °C/min); the DSC curves for the q^+^  = 2 °C/min; the DSC curves for the q^+^  = 5 °C/min; the DSC curves for the q^+^  = 20 °C/min.

**Fig. 2:**
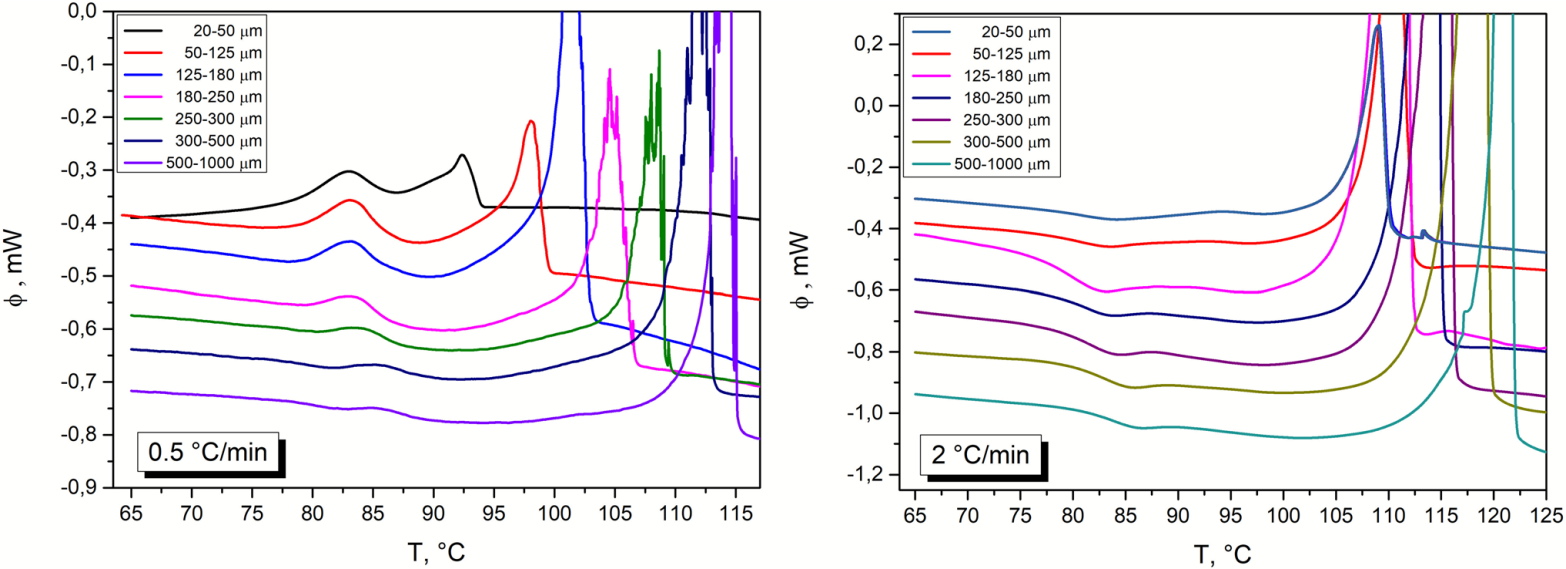
The detail of the obtained DSC curves for all studied particle size fractions at such low applied heating rates – chosen heating rates 0.5 and 2 °C/min

The basic DSC characterization (temperature dependences of the DSC signal, i.e., heat flow Φ) of the prepared amorphous griseofulvin powders (size fractions 20–50, 50–125, 125–180, 180–250, 250–300, 300–500, and 500–1000 μm) is shown in Figs. 1 and 2. The characteristic phenomena associated with the heating of the amorphous material can be identified in most of the DSC curves. In the case of griseofulvin, the weak endothermic step change representing the glass transition is found in the temperature range of 75–90 °C. At low heating rates (0.5–2 °C·min^−1^), the glass transition effect overlaps with a small exothermic peak corresponding to the partial crystallization (see Fig. 2 for the detailed view). This crystallization pre-peak is followed by a second exothermic effect (main crystallization event) between 95 °C and 135 °C (depending on q^+^). The endothermic melting peak is situated in the 200–220 °C range (extrapolated onset at 215 °C).

In Fig. 2, the zoomed-in glass transition and crystallization regions are shown for the curves measured at low q^+^. At 0.5 °C·min^−1^, the crystallization pre-peak practically always (except for the two coarsest powder fractions) fully overlaps with the glass transition effect. Considering the very weak evolution of T_g_ with particle size (known for higher q^+^—see Fig. 1 or Fig. 2B), it is clear that in the case of the finest griseofulvin powders, the crystallization process manifesting through the pre-peak initiates below T_g_. This finding suggests that the origin of this pre-peak could be associated with the diffusionless surface growth of crystals (so-called GC growth), which usually occurs in the pharmaceutical substances just below T_g_ [20] and initiates from mechanical defects. This unusual type of crystal growth is caused by the high surface mobility of certain low-molecular organic glasses (orders of magnitude higher compared to the bulk self-diffusion), which is below T_g_ not disrupted by the relaxation motions and general fluidity of the surface layers [20, 21]. During the diffusionless surface growth, the crystal formation continuously creates new free surfaces and voids due to the higher density of the crystal compared to the density of the amorphous phase. Thus, the surface mobility and crystallization are further accelerated. The crystallization of the griseofulvin causes a large density increase of up to 8% (in the case of the other amorphous substances, the density increase of 5% and less occurs), which leads to the marked acceleration of the below-T_g_ crystallization process (usually not observed macroscopically in the DSC data). Furthermore, the nucleation and crystal growth processes are (usually) accelerated in the presence of mechanical defects [22]. For the present data (namely the marked manifestation of the GC growth crystallization peak), the influence of both these phenomena is assumed.

**Fig. 3:**
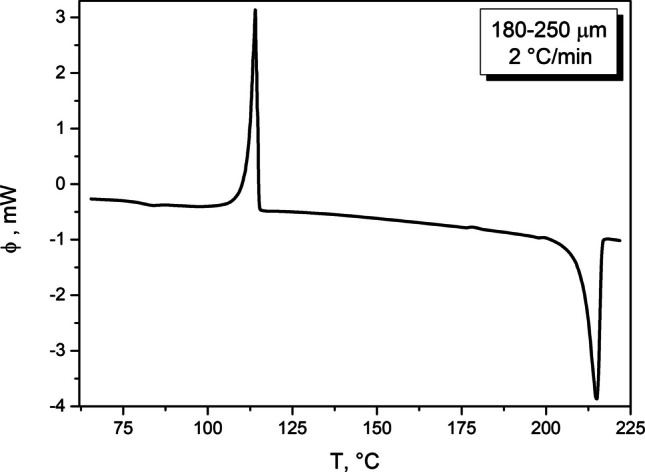
The single DSC curve measured for the 180–250 µm particle size fraction and applied heating rate q^+^  = 2 °C/min.

Regarding the main crystallization peak, it has a clearly negative asymmetry (skewing to higher T, with the onset peak edge more gradual compared to the sharp endset edge), which is rather typical for the non-catalyzed nucleation-growth or zero-order kinetic mechanisms—see Figs. 1 and 3. However, it cannot be ruled out that the crystallization process is more complex, consisting of several sub-processes corresponding to, e.g., the formation of different morphological, location-diversified, or polymorphic phases. Most polymorphs of griseofulvin can form from melt (i.e., their nucleation needs to be considered for the present case); crystal growth of form II is, however, significantly slower compared to the other crystalline forms [6].

**Fig. 4:**
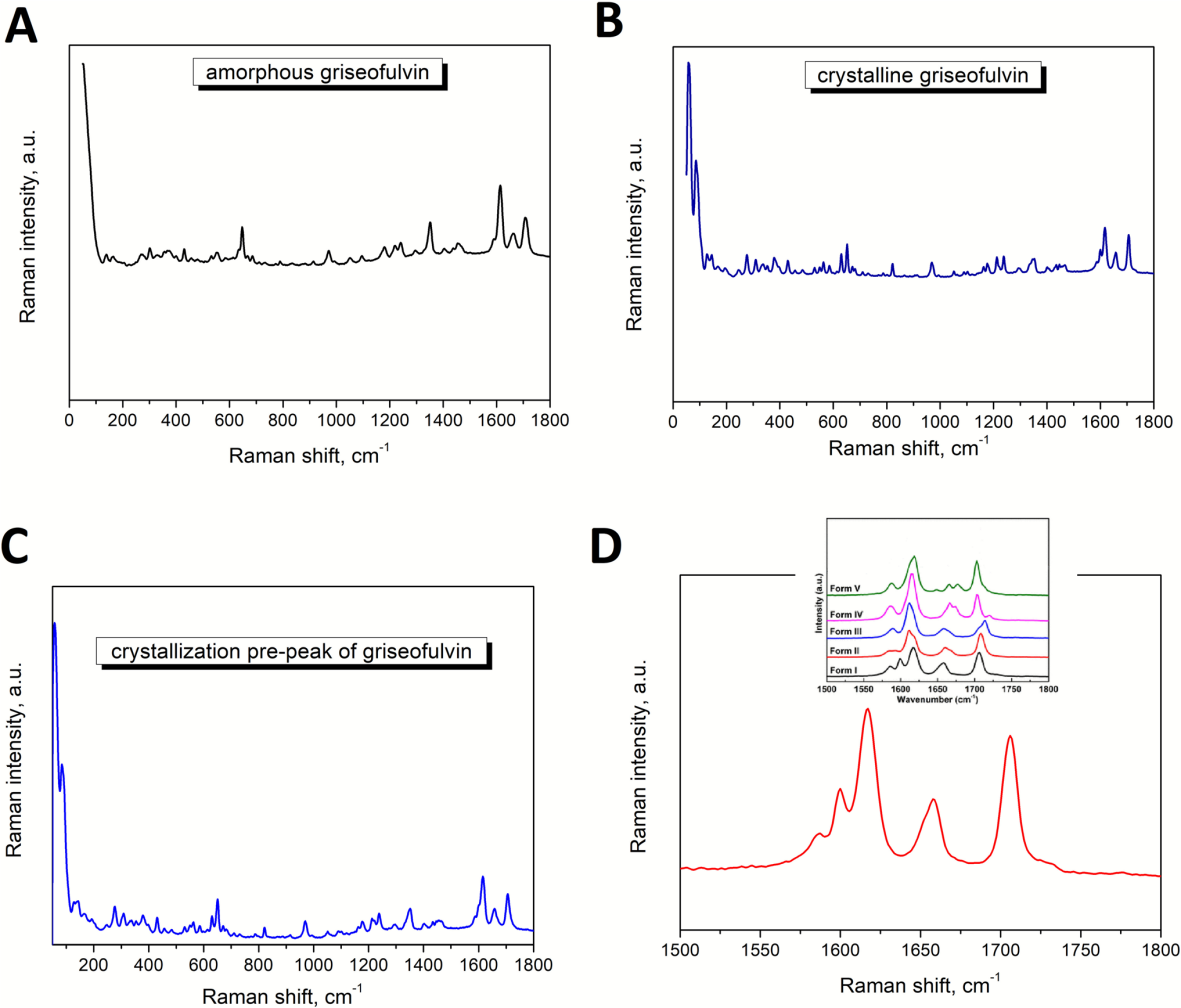
The Raman spectra – The obtained Raman spectrum for the amorphous sample of the griseofulvin (**A**) and for the crystallized sample of the griseofulvin (**B**); (**C**) – The Raman spectra of the griseofulvin measured after the removing the sample at a given moment from the DSC instrument (the partially crystallized sample to „the crystallization pre-peak “); (**D**) – The comparison of the Raman spectra for the polymorphs of the griseofulvin taken from the literature and the measured Raman spectrum of the crystallized griseofulvin.

Since different polymorphs of a given API can have a strong influence on the therapeutic properties of drugs (such as the chemical stability, including solubility, dissolution rate, and bioavailability [23]), their identification is crucial. In the present work, Raman spectroscopy was used in this regard. First, however, Raman spectroscopy was used to verify the amorphous character of the griseofulvin powders. As can be deduced from the comparison of Fig. 4A and B, the originally prepared griseofulvin powders were indeed fully amorphous, as the edge of the Rayleigh line (0–100 cm^−1^ spectral range) is smooth, without any bands. The comparison of Fig. 4B and C then testifies about the identical polymorphic nature of the crystalline phases formed during the GC and main crystal growth processes. Concerning the exact identification of the polymorphic phase, the comparison of the present Raman signal measured in the 1550–1750 cm^−1^ spectral range with the corresponding literature data for different GSF polymorphs shows that very probably only the dominant form I is present – see Fig. 4D.

#### Microscopy Characterization

**Fig. 5:**
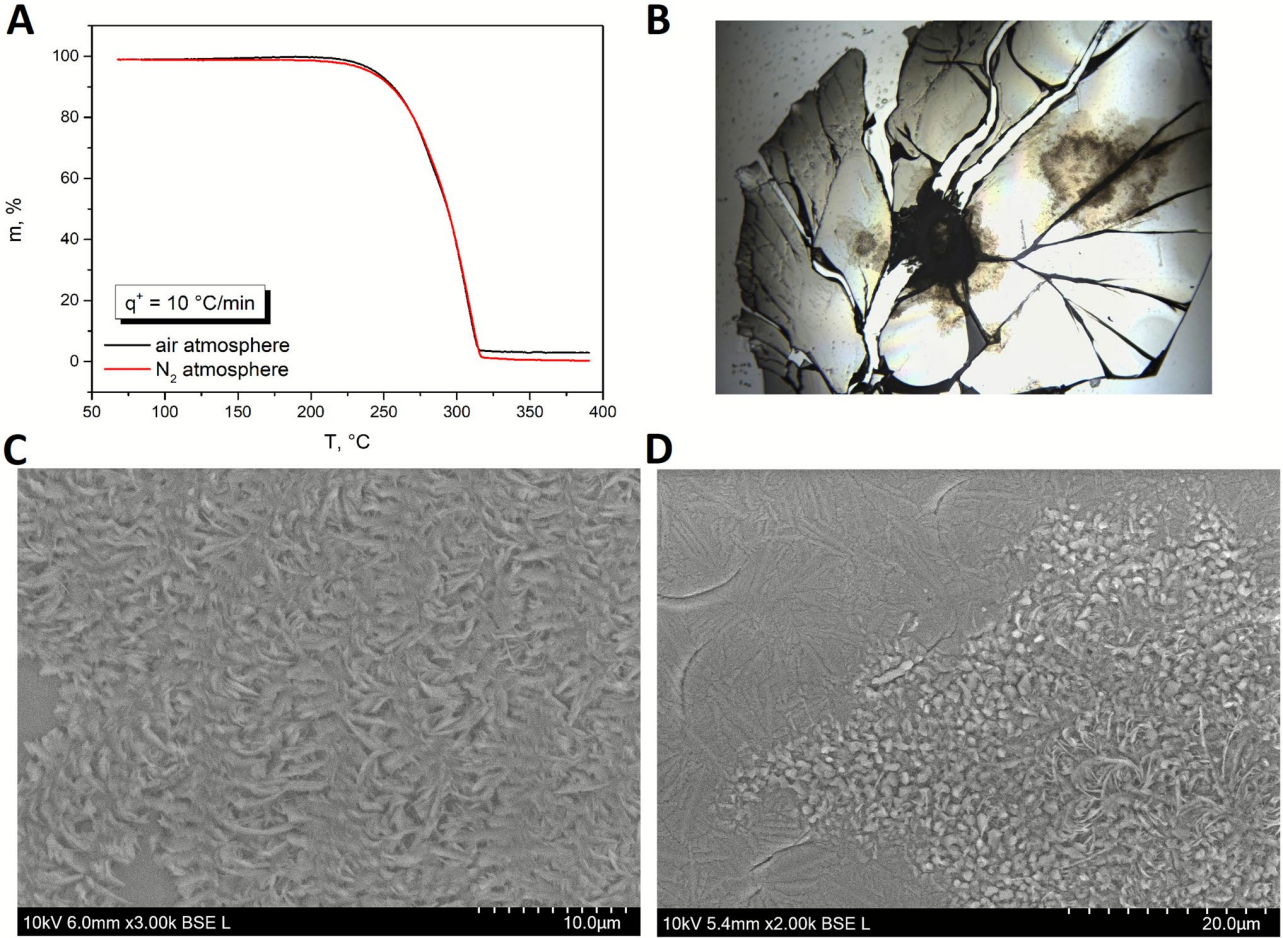
(**A**) The thermogravimetric curves of the griseofulvin measured under the air and N_2_ atmosphere; (**B**) The optical micrograph of the griseofulvin crystals growing at 75 °C from a microcrack; The SEM photos obtained for amorphous (**C**) and partially crystallized (**D**) sample of the griseofulvin.

In addition to the thermoanalytical and spectroscopic techniques, microscopies were used to examine the morphology of the formed crystalline phase. Figure 5B shows a micrograph of a long-term (~ 30 days) crystalized sample at laboratory temperature – the smooth surface of an amorphous droplet on a microscopic slide had to be slightly scratched to initiate the crystal growth. The dark areas in Fig. 5B represent the primary crystalline form formed from the damaged surface. The light brown shade represents a thin surface layer of the crystalline phase. The sharp and long edges of the surface crystalline phase indicate that the crystal growth propagates along the microcracks formed during scratching. The major cracks depicted in Fig. 5B are a consequence of the sample contraction griseofulvin has a much larger thermal expansion coefficient than the microscopic glass). These cracks appear only after the cooling due to the thermal expansion and disappear almost immediately after the heating above T_g_ (the material was repeatedly heated and cooled around the T_g_, and this process did not lead to any crystallization, probably because the cracks always disappeared immediately above the T_g_, hence to growth initiation occurred). As such, this phenomenon may be considered a form of self-healing [24–28].

The SEM micrographs are depicted in Fig. 5C (amorphous surface layer) and D (partially crystallized GSF—20 min at 90 °C). The differences between both studied samples are evident. Whereas the amorphous material is irregularly structured (interestingly, with a leaf-like surface morphology), the GSF crystalline phase is gritty, with the size of individual grains ~ 1–2 μm.

#### Thermogravimetric Characterization

Assessment of the high-T thermal stability of the GSF molecule was investigated using thermogravimetry. The weight loss curves obtained in the air and N_2_ atmospheres are shown in Fig. 5A; no significant water content was found even for the finest griseofulvin powder (the large surface-to-volume ratio is most prone to the physical adsorption of water molecules). Both weight loss curves are practically identical, with the first signs of the thermal decomposition being recognizable at T ≈ 200–210 °C (at 10 °C·min^−1^). It is noteworthy that in the air, complete gasification was not achieved, with a carbon residue of ~ 3—4% left in the aluminum crucibles. Apart from assessing the GSF stability during its melting and consequent quench-amorphization, the drugs’ thermal stability is particularly important for the nowadays popular application of pharmaceutical 3D printing, which is relevant for a majority of orally administered drugs as a path toward personalized medicine. Whereas the typical hot-melt extrusion temperatures for the pharmaceutically relevant biopolymers (e.g., hydroxypropylmethylcellulose) are 140–160 °C, the consequent 3D-printing from the API-containing filaments usually require higher temperatures (190–210 °C). In the case of griseofulvin, this is already a borderline for a relatively fast thermal degradation. Hence, close attention needs to be paid to the minimization of the time spent at these temperatures.

### Crystallization Kinetics of Amorphous Griseofulvin Powders

The DSC crystallization data obtained for the main crystallization peaks (occurring under all conditions) were analyzed by means of the standard methodology of solid-state kinetic analysis [29]. In particular, the raw DSC heat flow data were first treated in terms of the subtraction of the thermo-kinetic background – the physically meaningful tangential area-proportional baseline [30] was used to separate the crystallization signal from the underlying parasitic signal produced by the intrinsic heat capacity of the system. In this regard, Eq. 1 was used:

1 $$B\left(T\right)=\left(1-\alpha \left(T\right)\right)\cdot \left({z}_{0,r}+{z}_{1,r}\cdot T\right)+\alpha \left(T\right)\cdot \left({z}_{0,p}+{z}_{1,p}\cdot \left({T}_{f}-T\right)\right)$$

where *B*(*T*) is the temperature dependence of the baseline curve, *α* is the degree of conversion, *z*_*0,r*_*,* and *z*_*1,r*_ are the coefficients characterizing the tangent going through the starting point (in the reactants area), *z*_*0,p*_ and *z*_*1,p*_ are the coefficients characterizing the tangent going through the endpoint (in the products area), and *T*_*f*_ is the endpoint temperature.

The pure crystallization signal was consequently modeled within the framework of the basic solid-state Arrhenian kinetics expressed by Eq. 2 [29]:

2 $$\Phi=\triangle H_c \cdot A \cdot e^{-E_c/RT} \cdot f(a)$$

where Φ is the heat flow (measured by DSC), ΔH_c_ is the crystallization enthalpy, A is the pre-exponential factor, E_c_ is the apparent activation energy of the crystallization process, R is the universal gas constant (8.314 J·K^−1^ mol^−1^), T is temperature, α is the degree of conversion and f(α) is a mathematic function representing an appropriate kinetic model. The kinetic models utilized for the crystallization processes are essentially reduced to the physically meaningful nucleation-growth Johnson–Mehl–Avrami (JMA, Eq. 3) model [31–33] and the empirical autocatalytic Šesták-Berggren (AC, Eq. 4) model [29]:

3 $$f{\left(\alpha \right)}_{JMA}=n\left(1-\alpha \right){\left[-\mathrm{ln}\left(1-\alpha \right)\right]}^{1-\left(1/m\right)}$$

4 $$f{\left(\alpha \right)}_{AC}={\alpha }^{M}{\left(1-\alpha \right)}^{N}$$

where m, M, and N are the kinetic exponents responsible for the asymmetry and shape of the DSC crystallization peak. Due to the interpretation possibility of the kinetic exponent m, the JMA model is preferred – a quantified approach to the testing of the JMA model applicability was recently introduced in the reference [34]. The enumeration of Eq. 1 is usually split into two procedures – model-free (determination of E_c_ and estimation of A) and model-based (determination of the appropriate reaction mechanism with the suitable kinetic model functions f(α) and their parameters, calculation of the corresponding Δ*H*_*c*_, and refinement of A). In the first step, the Kissinger method [35] (peak-based method; Eq. 5), Friedman method [36] (differential isoconversional method; Eq. 6), and Starink method [37] (integral isoconversional method; Eq. 7) were used to cover the standard methodological approaches [38, 39] (integral isoconversional method; Eq. 7) were used to cover the standard methodological approaches.

5 $$\mathrm{ln}\left(\frac{{q}^{+}}{{T}_{p}^{2}}\right)=-\frac{{E}_{c}}{R{T}_{p}}+const.$$

6 $$\mathrm{ln}\left({\left[d\alpha /dt\right]}_{\alpha }\right)=-\frac{E}{R{T}_{\alpha }}+const.$$

7 $$\mathrm{ln}\left(\frac{{q}^{+}}{{T}_{\alpha }^{1.92}}\right)=-1.008\frac{E}{R{T}_{\alpha }}+const.$$

**Fig. 6:**
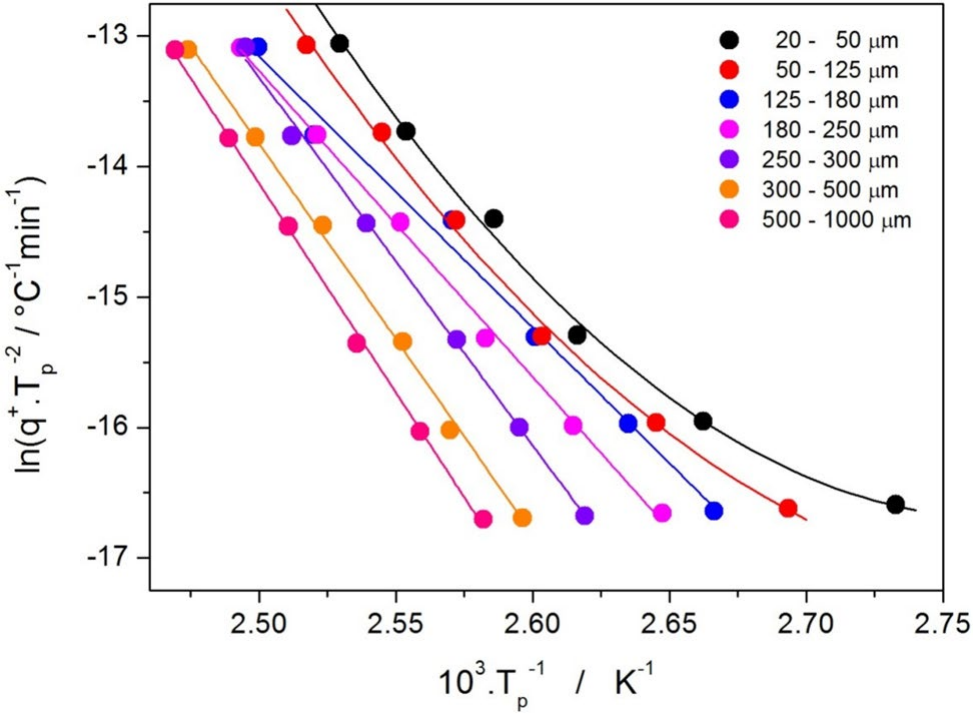
Kissinger plot (based on Eq. 5) for the crystallization of amorphous griseofulvin powders. Lines indicate either polynomial (20–50 μm and 50–125 μm powders) or linear (powder fractions from 125–180 μm up) fits of the data.

In these equations, the T_p_ corresponds to the maximum of the crystallization peak; (dα/dt)_α_, T_α_ and E_α_ are the conversion rate, temperature, and activation energy corresponding to arbitrarily chosen values of conversion α. Since the cold crystallization in the glasses (crystallization occurring during heating of the amorphous state) proceeds, if exhibiting a kinetic complexity, via reaction mechanism consisting of independent processes, the Kissinger method was shown [40] to be particularly useful for determining E_c_ of the main/dominant crystallization process. The Kissinger plot for the present data (main crystallization peak of the different amorphous griseofulvin powders) is shown in Fig. 6.

**Fig. 7:**
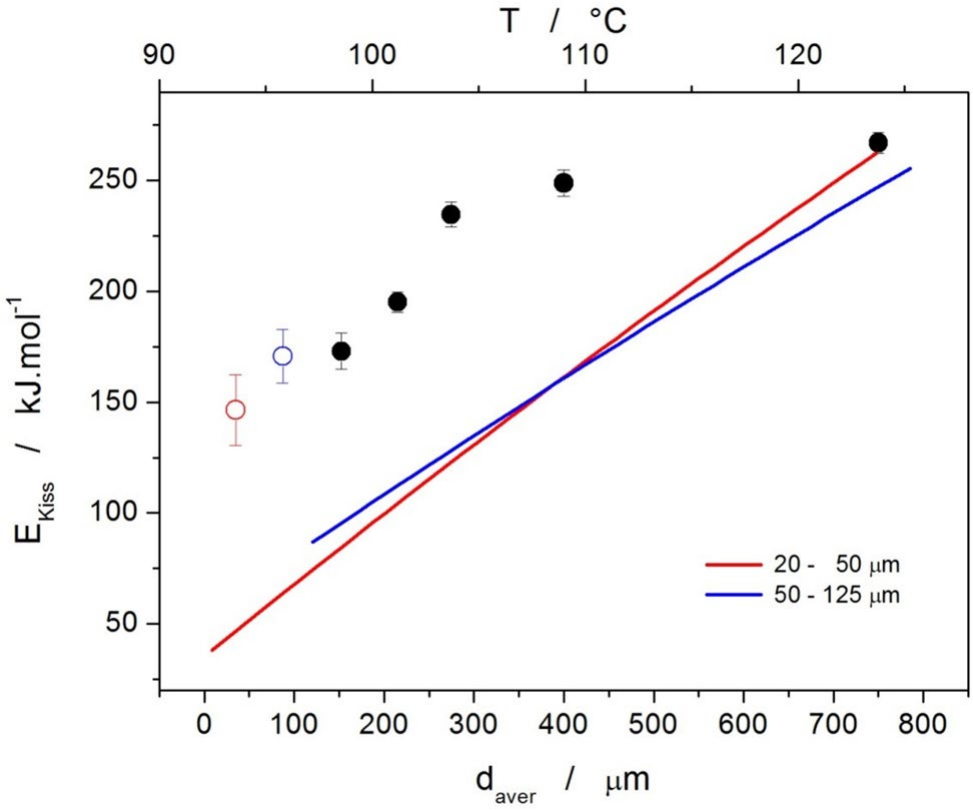
Activation energies determined by the Kissinger method for the crystallization of amorphous griseolfulvin powders. Points (attributed to the left and bottom axes) indicate the E_Kiss_ values determined from the linear fits of the dependences depicted in Fig. 6. Lines (attributed to the left and top axes) indicate the E_Kiss_-T dependences obtained from the derivation of the polynomial fits of the two finest griseofulvin powders.

As is apparent from Fig. 6, the coarse powder fractions exhibited good linearity of the Kissinger dependences; the two finest powders showed a significant curvature at the lowest heating rates (probably due to the enhanced nucleation and/or sub-T_g_ crystal growth proceeding during the DSC measurement itself), shifting the crystallization data to lower temperatures, and accelerating the overall crystallization process. This is also apparent from Fig. 1. For this reason, the Kissinger data for these two finest powder fractions were fit by the second-order polynomial functions (contrary to the linear fits of the data for coarser powders), and the corresponding temperature dependences of E_c_ were estimated from the derivation of these polynomial fits. The resulting E_c_ values (denoted E_Kiss_ to indicate the method of their determination) are for the studied griseofulvin powders shown in Fig. 7.

Interestingly, the data obtained for the two finest powder fractions indicate a switch behavior (compared to the next larger powder fraction), where at high q^+^, the activation energy is similar to that of the bulk/coarsest powders, and with decreasing q^+^ (possibly mostly relevant with regard to the longer duration of the sub-T_g_ nucleation and growth stage), E_Kiss_ decreases to very low values that indicate rapid acceleration of the growth process. Note that for the powders with d_aver_ ≥ 125 μm appear to average the two extreme types of the crystallization behavior throughout the whole range of applied q^+^. This indicates that at least two different nucleation/growth mechanisms occur at higher q^+^. Considering the tendency of the low-molecular organic glasses to crystallize from the surface defects [41, 42], the most probably involved mechanisms are as follows: above-T_g_ surface crystal growth from mechanical defects (micro-cracks, edges); above-T_g_ volume crystal growth from internal mechanical defects; secondary above-T_g_ crystal growth continuing from the crystallization centers formed via the below-T_g_ rapid glass-crystal growth mechanism [42]. From a practical point of view, the crystal growth appears to have slightly ceased at higher q^+^ for the middle-sized powder fractions. Akin behavior was recently reported for the amorphous indomethacin [43]. It is also important to notice that the shape of the crystallization peaks does not significantly change (no splitting of the dominant crystallization peak is observed) with q^+^ or d_aver_, which indicates that E_Kiss_ can be used as a good approximation of the overall crystallization behavior (including the potentially occurring minor/secondary crystal growth processes) [38–40, 44].

In addition to the Kissinger method, the two isoconversional methods were used, determining the E_c_-α dependences for the present crystallization data. Generally, the isoconversional results confirm the findings of the Kissinger evaluation. Whereas in the case of the present data, the isoconversional analysis does not add any new information and only supports the results obtained via the Kissinger equation, the course of the E-α dependences can still be of interest to certain readers – it is therefore included (together with a brief commentary) in the Supplemental online material.

**Fig. 8:**
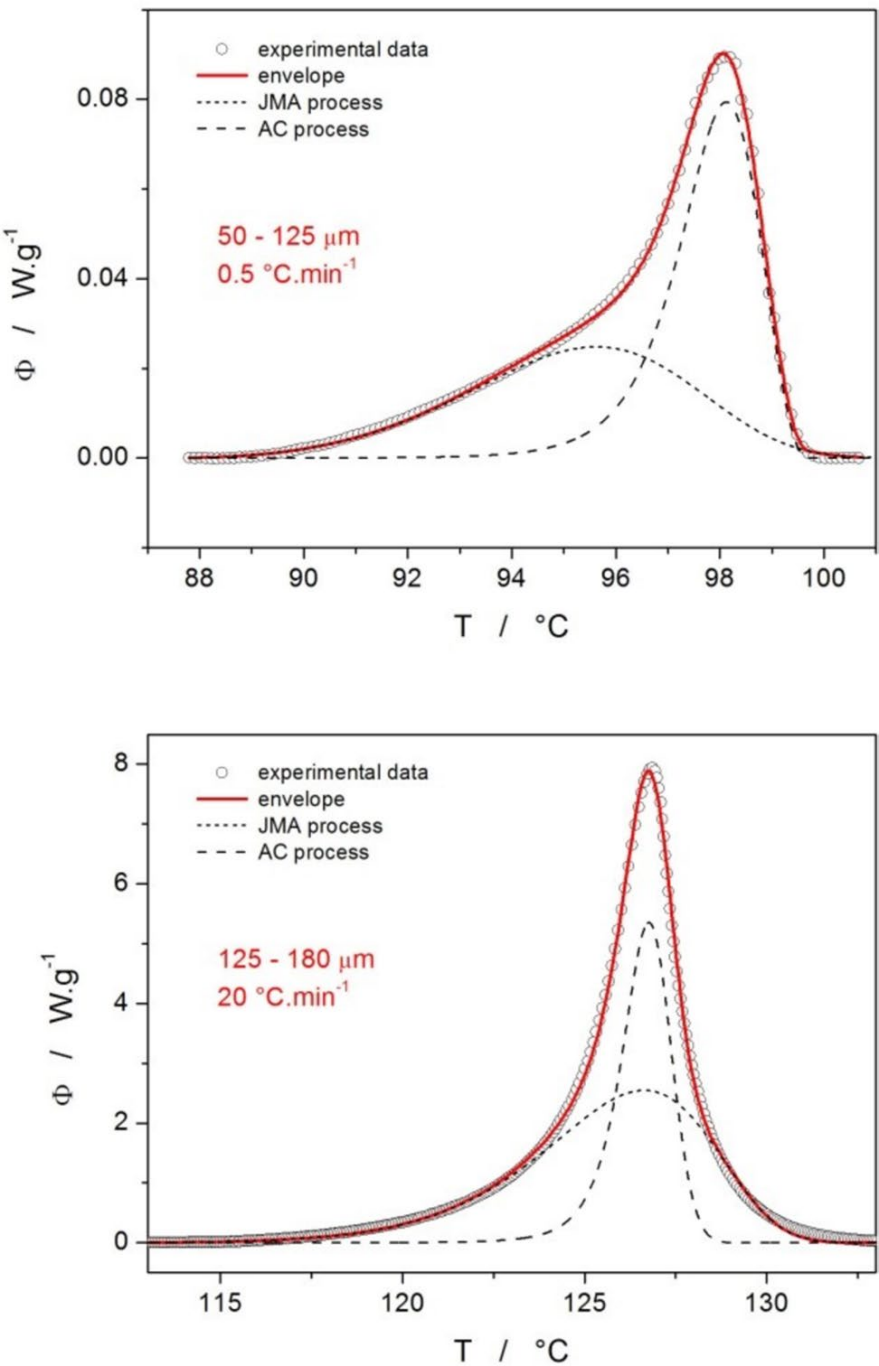
Example sc-MKA fits of the DSC crystallization data obtained for the amorphous griseofulvin

With the knowledge of E_c_, the model-based kinetic analysis can be performed by means of the sc-MKA (single-curve multivariate kinetic analysis) approach [45]:

8 $$RSS=\sum_{j=1}^{n}\sum_{k=Firs{t}_{j}i}^{Las{t}_{j}}{w}_{j,k}{\left(Y{\mathrm{exp}}_{j,k}-Yca{l}_{j,k}\right)}^{2}$$

9 $${w}_{j}=\frac{1}{{\left|{\left[d\alpha /dt\right]}_{\mathrm{max}}\right|}_{j}+{\left|{\left[d\alpha /dt\right]}_{\mathrm{min}}\right|}_{j}}$$

where RSS is the sum of squared residue, n is number of measurements, j is index of the given measurement, First_j_ is the index of the first point of the given curve, Last_j_ is the index of the last point of the given curve, Yexp_j,k_ is the experimental value of the point k of curve j, Ycal_j,k_ is the calculated value of the point k of curve j and w_j_ is weighting factor for curve j. Note that during sc-MKA, E_c_ needs to be fixed at a single value for each optimized curve – for this purpose, the E_Kiss_ values (including the E_Kiss_-T dependences for the two finest powders) were used. The Y_cal_ quantity was modeled in terms of several different reaction mechanisms; the simplest satisfactory results were obtained for the combination of two independent processes, one with JMA (Eq. 3) and one with AC (Eq. 4) kinetics. In particular, the following equations were used for the sc-MKA optimization:

10 $$\Phi=\Delta H_1{\cdot A}_1 \cdot e^{E_1/RT}n\cdot\left(1-\alpha\right)\cdot\left[-ln\left(1-\alpha\right)\right]^{1-\left(1/n\right)}+\Delta H_2{ \cdot A}_2 \cdot e^{E_2/RT} \cdot \alpha^M\left(1-\alpha\right)^N$$

11 $$\alpha =\frac{\Delta {H}_{1}}{\Delta H}{\alpha }_{1}+\frac{\Delta {H}_{2}}{\Delta H}{\alpha }_{2}$$

where the indices "1" and "2" indicate the first and second processes, respectively. Examples of these fits performed for the data measured at low and high q^+^ are shown in Fig. 8; this archetypal behavior was shared across all measured particle size fractions, regardless of d_aver_. The individual sets of kinetic parameters obtained by means of the sc-MKA method for the individual DSC curves are listed in the Supplemental online material.

The q^+^-dependent mutual positioning of the two kinetic peaks (including the overall initiation with the JMA kinetics) was identified for all samples, despite certain random variations in the peaks’ magnitude, minor changes of their asymmetry, and DSC signal scatter associated with individual (rather than averaged) manifestation of the growth processes arising from scarce crystallization centers in the case of coarse powders.

Above, it was shown that decreasing the GSF particle size massively increases its tendency towards crystallization due to the high free energy of the grain surface paired with the presence of mechanical defects. In the following section, the practical implications of this fact will be explored in regard to the stability of amorphous GSF particles incorporated into a standard pharmaceutical matrix tablet for controlled release, which will be subject to the dissolution tests. Since the high free surface energy of the amorphous phase primarily accelerates the dissolution, the large surface with high amounts of defects may prove to be detrimental due to the negative effect on the stability of the amorphous phase during the dissolution. In addition, the magnitude of the impact of the particle size on the dissolution rate (considered in terms of the Noyes-Whitney relation) will be explored by comparison with the dissolution characteristics of the original as-purchased crystalline GSF.

### Dissolution Behavior of Hydrophilic Tablets With Amorphous and Crystalline Griseofulvin

**Fig. 9:**
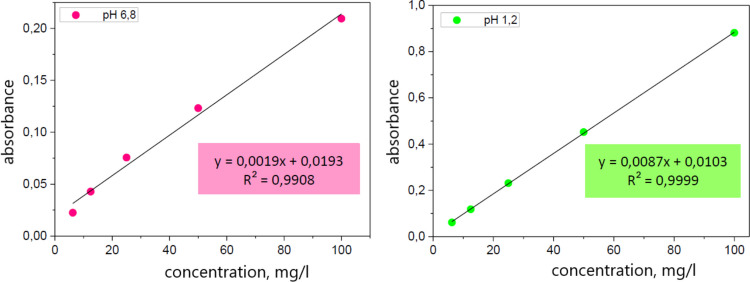
The calibration curves of the hydrophilic tablets containing the griseofulvin; left for the dissolution medium of pH 1.2; on the right for the dissolution medium of pH 6.8; stated regression equations to the calculation of the absorbance to the GSF concentration

**Fig. 10:**
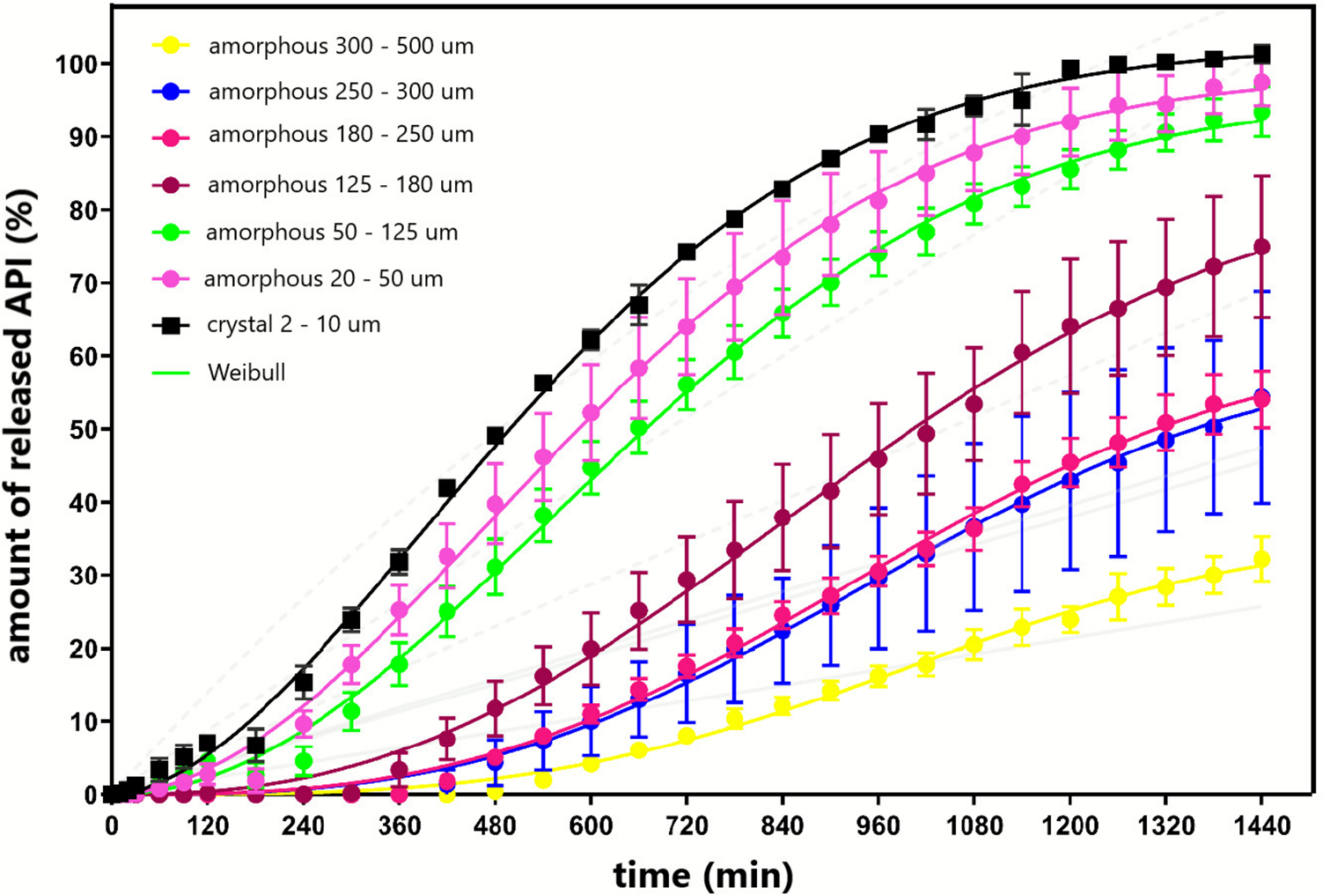
The dissolution profile of the hydrophilic tablets containing griseofulvin for the crystal and the given amorphous particle size fractions of GSF fitted to the Weibull model

In addition to the solid-state characterization of the griseofulvin powders, the influence of the particle size and crystallinity on the dissolution behavior of the prepared hydrophilic tablets containing the amorphous or crystalline griseofulvin was studied by means of a dissolution test. To obtain the dissolution profile, first, the calibration curve from the prepared calibration solutions must be determined, i.e., the dependence of the absorbance on the concentration of the drug for pH 1.2 and pH 6.8 needs to be determined. Using the linear regression method, the relationships highlighted in Fig. 9 were obtained. Using these equations, the experimental absorbance values obtained during the dissolution tests were converted to the percentage of the released drug. The resulting dissolution profiles are shown in Fig. 10 for the tablets containing the different prepared amorphous powders as well as for the set of tablets containing the original as-purchased crystalline GSF. The quantitative evaluation was performed using the non-linear regression analysis method in the GraphPad Prism® program; the experimental data were fit to the Weibull model (see Eq. 12):

12 $${M}_{t}={M}_{\infty }(1-\mathrm{exp}\left(-{k}_{W}{t}^{\beta }\right))$$

where M_t_ is the amount of the drug released in the time t, M_∞_ is the maximum amount of the drug that can be released from a dosage form in the infinite time, k_w_ is the constant of the Weibull model, the parameter β stands for the shape of the exponential curve [46].

As is commonly stated in literature (see, e.g., [11, 13]), the amorphous phase should exhibit a significantly higher solubility compared to the crystalline form of API. However, in the present case of griseofulvin, the very finely grained (particle size ~ 2–10 μm) crystalline GSF, which was incorporated into the hydrophilic tablet, dissolved much faster compared to the finest prepared amorphous GSF powder. Considering the very large difference in the isochronal values of released API observed for the different particle size fractions of amorphous griseofulvin, it has to be concluded that (for this API), the solubility of the crystalline and amorphous forms is roughly similar, and the dominant role is played by the size of the individual compact API grains: the larger the grain, the lower the surface/volume ratio, and the slower the dissolution process driven by the diffusion through the interface between the solid grain surface and the dissolution medium. As the physico-chemical principle connecting the dissolution rate and particle size is dictated by the Noyes-Whitney relation, the approx. 10 × larger surface area of the crystalline powder (compared to that of the finest amorphous powder) clearly outweights the impact of the increased intrinsic solubility (and also possibly higher diffusion coefficient through the solid–liquid interface) associated with the amorphous phase. Note that the estimate of the ratio between the two surface areas was done based on the average particle size (calculating the surface/volume ratio for the lowest particle diameter values as they are always the most represented) and the assumption of an akin degree of non-sphericity between the two considered powders. Another reason, why the absolute difference between the intrinsic solubilities of the crystalline and amorphous phases is only modest, may be the BCS classification of GSF into the class II associated with overall low aqueous solubility. It is clear from Fig. 10 that only around 30% of the griseofulvin was released during 24 h from the tablet containing the amorphous griseofulvin with a size fraction of 300–500 μm. Only the tablets that contained crystalline (2–10 μm) and the amorphous griseofulvin with a size fraction of 20–50 μm reached ~ 100% API release within 24 h. The solubility of the studied API in both dissolution media at 37 °C was experimentally verified. In the acidic dissolution medium (pH 1.2), the solubility was determined to be 0.06 mg/ml, and at pH 6.8 it was 0.07 mg/ml.

**Fig. 11:**
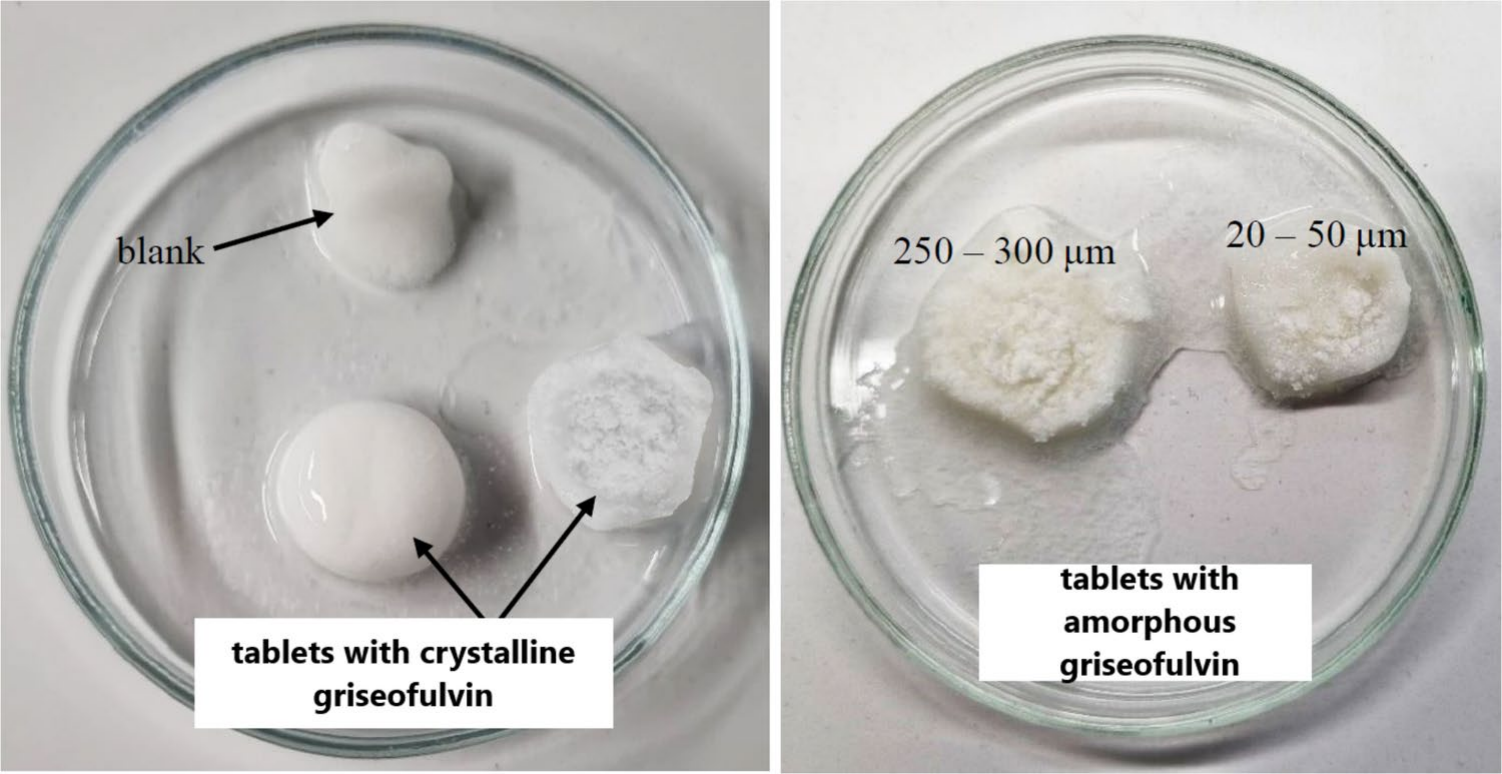
The tablets after the completion of the dissolution test. The top left is the blank tablet, and below the crystalline griseofulvin tablets, on the right – are the tablets with the amorphous griseofulvin (size fractions 250–300 μm and 20–50 μm)

The hydrophilic tablets usually swell due to the absorption of the dissolution medium – see Fig. 11. The swelling results in a loosening of the polymer chains in the tablet and the release of the API from the tablet by a diffusion process. During this swelling, the gel layer of the tablet is formed, into which the active substance from the core of the tablet enters. The gel layer, together with the API, is washed off with the constant formation of another gel layer and the subsequent washing off. If the API is poorly soluble, the retention of the active substance inside the tablet could occur. The tablets in the left-side micrograph contained the crystalline griseofulvin – despite the 100% API release, the remaining undissolved tablet matrix (excipients) is clearly consistent, retaining the tablet’s original shape. The tablets in the right-side micrograph contained the amorphous griseofulvin. The tablet containing the 20–50 μm amorphous GSF powder released practically all API during the dissolution; thus, only auxiliary excipients form the depicted tablet residuum. On the other hand, only approx. 55% of API was released from the tablet containing the 250–300 μm GSF powder. Since no remaining yellowish (characteristic color of amorphous griseofulvin) API grains are apparent in the tablet residuum (nor were they found after cutting the residuum into small pieces), it can be either assumed that the remaining API physically interacted with the matrix tablet gel (and the remaining small grains are tightly covered with the white gel substances) or that a thin crystalline layer formed on the grains’ surface during the dissolution (Table I).

**Table I:** The Summary of the Used Substances and Their Amount in Prepared Tablets for the Dissolution Tests

Substance	Amount, mg
Griseofulvin	50
Prosolv® SMCC 90	245
Hypromellose K4M	200
Magnesium stearate	5

**Fig. 12:**
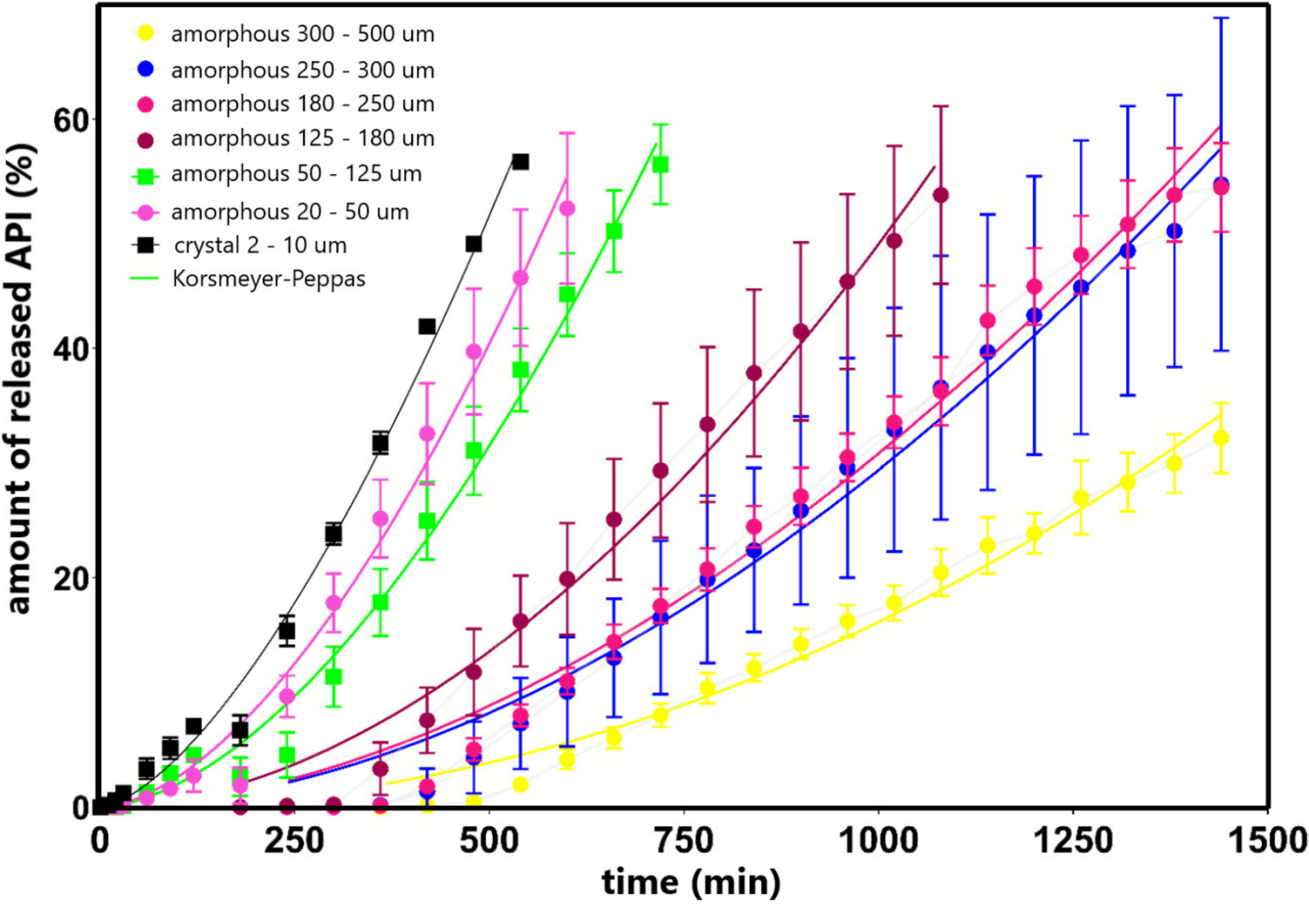
The dissolution profile up to 60% of the released griseofulvin fitted to the Korsmeyer-Peppas model

To estimate the release mechanism of griseofulvin from the prepared tablets, the dissolution profiles were fit to the Korsmeyer-Peppas model—see Fig. 12 and Eq. 13. Note that this model is valid up to the 60% drug release. Hence, the corresponding fitting of the experimental data. The values of the model parameter n determined for each studied size fraction are listed in Table II. The parameter n characterizes the transport mechanism of the drug from the dosage form. With regard to the present values, the case of n > 1 is relevant, which indicates that the drug is released by the so-called super case II transport, which occurs for swelling polymers, where diffusion and erosion are combined [47]. The gradually increasing n values with the rising size of the API particles incorporated into the tablets indicate that the API release is indeed slowed down, characterized by a decreasing initial slope of the dissolution profile onset.

**Table II:** The Values of the Parameter N of the Korsmeyer-Peppas Model for the Given Size Fractions of the Studied Griseofulvin

size fraction of griseofulvin	parameter n ± SD
crystal 2–10 µm	1.53 ± 0.05
amorphous 20–50 µm	1.69 ± 0.09
amorphous 50–125 µm	1.70 ± 0.07
amorphous 125–180 µm	1.85 ± 0.12
amorphous 180–250 µm	1.80 ± 0.6
amorphous 250–300 µm	1.84 ± 0.17
amorphous 300–500 µm	2.06 ± 0.08

13 $$\frac{{M}_{t}}{{M}_{\infty }}={k}_{KP}{t}^{n}$$

In Eq. 13, the kKP stands for the constant of the Korsmeyer-Peppas model, and n stands for the parameter n.

## Conclusions

In the first part of the reported research, the crystallization of amorphous GSF powders was studied in dependence on particle size. It was shown that fine GSF powders (with large amounts of surface mechanically induced defects) are particularly prone to the rapid diffusionless GC crystal growth, which characteristically occurs for low-molecular organic glasses with high surface mobility (self-diffusion) below T_g_. The specificity of GSF lies in the major dominance of this growth mode, which can (atypically) proceed up to ~ T_g_ + 62 °C and further increases the risk of amorphous phase degradation [48]. Since the GC growth is initiated by the protrusion along the micro-cracks and mechanical defects, the dominance of this growth mode increases with decreasing q^+^, which is associated with longer time spent in the critical temperature region: approx. from (T_g_ – 20 °C) to (T_g_ + 20 °C) [49]. Detailed kinetic analysis of the crystallization data has shown that, despite only the thermodynamically stable griseofulvin polymorph no. I being formed (confirmed by Raman spectroscopy), the crystallization processes are complex, which indicates multiple locations and/or growth mechanisms (supported by microscopic investigation). Moreover, the activation energy determined for the finest powder fractions shows that at low q^+^, the GC crystal growth gets progressively accelerated, which further increases the risk of the amorphous-to-crystalline transformation occurring at temperatures well below T_g_ (even close to the laboratory temperature) during long-term storage.

In the second part of the reported research, the dissolution behavior of the different amorphous GSF powder fractions incorporated in a pharmaceutical tablet was studied by means of the standard dissolution test and described in terms of the Korsmeyer-Peppas model (indicating the release by super case II transport). Whereas the actual physical state of the drug (amorphous *versus* crystalline) was found to be of low importance for the resulting dissolution rate, the particle size was confirmed to be absolutely crucial, controlling the released amount of the drug and shifting the dissolution profile by up to 10 h (in agreement with the Noyes-Whitney relation). It is also important to stress that despite the largely increased tendency toward crystallization confirmed for the fine amorphous GSF powders (Section "Crystallization kinetics of amorphous griseofulvin powders".), the amorphous form is (at least partially) stabilized in the present Hypromellose tablets, and do not show any significant compensation effect reducing the dissolution rate due to the formation of a crystalline layer on the surface of the fine amorphous grains (as evidenced by Fig. 10). This opens up an interesting possibility of fine-tuning the rate of dissolution based on the size of the used GSF powder. Note that the amorphous phase itself still has several advantages over using the crystalline powders – the amorphous powders are prepared more easily in different sizes (compared to their crystalline counterparts), and an introduction of partial crystallinity (e.g., crystalline surface layer with amorphous core) could provide further options for adjusting the dissolution profile to the patient’s needs.

## Data Availability

The data that support the findings of this study are openly available in FIGSHARE at https://figshare.com/s/c14779495c64aaba811a.
